# Mode of delivery and birth outcomes before and during COVID-19 –A population-based study in Ontario, Canada

**DOI:** 10.1371/journal.pone.0303175

**Published:** 2024-05-10

**Authors:** Teresa To, Jingqin Zhu, Emilie Terebessy, Cornelia M. Borkhoff, Andrea S. Gershon, Tetyana Kendzerska, Smita S. Pakhale, Nicholas T. Vozoris, Kimball Zhang, Christopher Licskai

**Affiliations:** 1 Dalla Lana School of Public Health, University of Toronto, Toronto, Ontario, Canada; 2 Child Health Evaluative Sciences, The Hospital for Sick Children, Toronto, Ontario, Canada; 3 ICES, Ontario, Canada; 4 Division of Paediatric Medicine and the Paediatric Outcomes Research Team (PORT), The Hospital for Sick Children, Toronto, Ontario, Canada; 5 Sunnybrook Health Sciences Centre, Toronto, Ontario, Canada; 6 Department of Medicine, The Ottawa Hospital and Ottawa Hospital Research Institute, University of Ottawa, Ottawa, Ontario, Canada; 7 Faculty of Medicine, Department of Medicine, University of Ottawa, Ottawa, Ontario, Canada; 8 Li Ka Shing Knowledge Institute, St. Michael’s Hospital, Toronto, Ontario, Canada; 9 Schulich School of Medicine and Dentistry, Western University, London, Ontario, Canada; Kobe University Graduate School of Medicine School of Medicine, JAPAN

## Abstract

There is lack of clarity on whether pregnancies during COVID-19 resulted in poorer mode of delivery and birth outcomes in Ontario, Canada. We aimed to compare mode of delivery (C-section), birth (low birthweight, preterm birth, NICU admission), and health services use (HSU, hospitalizations, ED visits, physician visits) outcomes in pregnant Ontario women before and during COVID-19 (pandemic periods). We further stratified for pre-existing chronic diseases (asthma, eczema, allergic rhinitis, diabetes, hypertension). Deliveries before (Jun 2018–Feb 2020) and during (Jul 2020–Mar 2022) pandemic were from health administrative data. We used multivariable logistic regression analyses to estimate adjusted odds ratios (aOR) of delivery and birth outcomes, and negative binomial regression for adjusted rate ratios (aRR) of HSU. We compared outcomes between pre-pandemic and pandemic periods. Possible interactions between study periods and covariates were also examined. 323,359 deliveries were included (50% during pandemic). One in 5 (18.3%) women who delivered during the pandemic had not received any COVID-19 vaccine, while one in 20 women (5.2%) lab-tested positive for COVID-19. The odds of C-section delivery during the pandemic was 9% higher (aOR = 1.09, 95% CI: 1.08–1.11) than pre-pandemic. The odds of preterm birth and NICU admission were 15% (aOR = 0.85, 95% CI: 0.82–0.87) and 10% lower (aOR = 0.90, 95% CI: 0.88–0.92), respectively, during COVID-19. There was a 17% reduction in ED visits but a 16% increase in physician visits during the pandemic (aRR = 0.83, 95% CI: 0.81–0.84 and aRR = 1.16, 95% CI: 1.16–1.17, respectively). These aORs and aRRs were significantly higher in women with pre-existing chronic conditions. During the pandemic, healthcare utilization, especially ED visits (aRR = 0.83), in pregnant women was lower compared to before. Ensuring ongoing prenatal care during the pandemic may reduce risks of adverse mode of delivery and the need for acute care during pregnancy.

## Introduction

Despite the increasing availability of literature on COVID-19 and pregnancy, there remains a lack of clarity around whether pregnancy during the COVID-19 pandemic resulted in poorer health, pregnancy, and newborn outcomes. Preterm labor, preeclampsia, and eclampsia were significantly higher in women with COVID-19 and underlying diseases like asthma, compared to those without underlying diseases [1]. Reviews also found that rates of Caesarean section (C-section) were higher in women with suspected, or confirmed, COVID-19 [2,3]. However, there was a lack of clinical reasons or etiology to help explain this C-section phenomenon. For example, Boukoura et al. concluded that the observed higher C-section percentages among women with COVID-19 may be attributable to COVID-19-stigma, risk management culture, and inadequate support of vagina birth [2]. Moreover, Di Toro et al. found that very few studies reviewed had reported COVID-19-related maternal complications as indications for C-section delivery, thus other clinical and non-clinical factors must have been considered when opting for C-section, despite guidelines and expert recommendations suggesting opting for vaginal deliveries whenever possible [3].

The experiences of pregnant women and reduced antenatal care during the COVID-19 pandemic differed across the globe. Missing antenatal clinic care was reported in low- and middle income countries (e.g., Saudi Arabia, 4] Sub-Saharan Africa, [5] India, [6] Pakistan [7]) and almost all middle-income countries [8]. Despite the reduced access to care, pregnant women in these countries were highly satisfied with the services provided by field workers. On the other hand, results from an online international cross-sectional survey with participants from high income countries (USA, Ireland and the UK) [9] suggested the lack of access to antenatal care coupled with reduced family/social support potentially intensified pregnancy specific stress. It was shown in Canada that during the COVID-19 pandemic, all-cause health services, including antenatal care, declined dramatically, particularly during the lockdowns [10,11]. The decline may partially be attributed to physicians helping patients avoid the emergency department (ED) or practicing more cautious medicine to avoid complications. However, the impact on pregnancy outcomes associated with declining health services was not well documented.

The objectives of this study were to use Ontario population-based administrative data to examine pregnant women’s differences of health services use (HSU), mode of delivery involving C-sections, and birth outcomes of women’s babies born before and during the COVID-19 pandemic. Birth outcomes include low birthweight, pre-term birth, and admission to neonatal intensive care unit (NICU) of babies at birth.

## Materials and methods

### Study population

**Fig 1:**
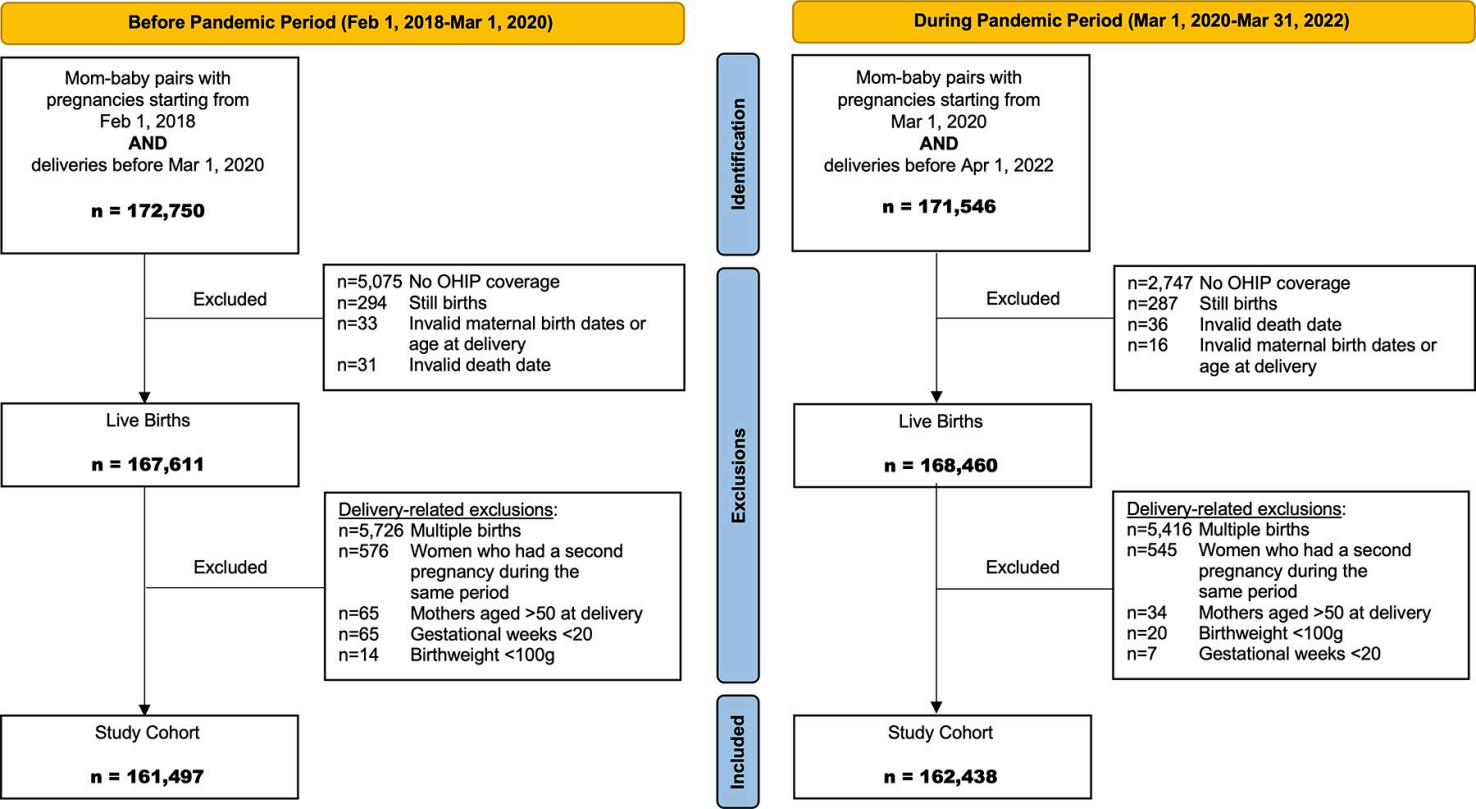
Study cohort identification flowchart.

This was a retrospective longitudinal cohort study in Ontario, Canada. Deliveries were identified from the provincial MOMBABY database housed at ICES (formerly the Institute for Clinical Evaluative Sciences). MOMBABY is an ICES-derived cohort linking maternal hospital delivery and newborn birth records. We included women aged 13–50 years at delivery from June 2018 to March 2022. Women with invalid date of birth, age at delivery, date of death, and those without Ontario Health Insurance Plan (OHIP) coverage during their pregnancy and/or delivery were excluded in this study. Additionally, deliveries of <20 weeks of gestation, stillbirths, birthweight <100 grams, and multiple births were excluded. Only the first pregnancy was included for women who had more than one pregnancy/delivery in each of the pre-pandemic and pandemic periods. See Fig 1 for more details on the study cohort identification.

### Data sources

This study used health administrative data that are routinely collected in Ontario. Five databases, in addition to MOMBABY, were used and linked: 1) Hospital admissions were captured by the Canadian Institute for Health Information Discharge Abstract Database (CIHI-DAD), 2) emergency department (ED) visits were captured by the National Ambulatory Care Reporting System (NACRS), 3) outpatient physician office and virtual visits from the OHIP claims database, 4) hospital admissions for mental health conditions were captured by the Ontario Mental Health Reporting System (OMHRS), 5) demographic information was captured by the Registered Persons Database (RPDB), 6) COVID-19 vaccination data from the provincial COVaxON Vaccination Management System, 7) COVID-19 testing results from the C19INTRG (COVID-19 Integrated Testing Database), and 8) Immigration status was captured by the Immigration, Refugees, and Citizenship Canada’s Permanent Resident Database (CIC).

### Exposure and outcome definitions

The primary exposures are the study periods: pre-pandemic versus pandemic.

*Study periods*: In Ontario, confirmed COVID-19 cases emerged as of March 2020. Therefore, in this study, we considered pregnancies and their respective deliveries occurring between June 2018 and February 2020 (21 months) as “*pre-pandemic*”. Pregnancies occurring from March 2020 onwards, whereby their respective deliveries including premature births that happened at earliest, between July 2020 and March 2022 (21 months), were considered “*pandemic*” deliveries. Pregnancies that happened in the pre-pandemic period (i.e., before March 2020) with their respective deliveries occurring in the pandemic period (i.e., after March 2020) were not included in this study as the exposure and outcomes crossed two study periods.

*One mode of delivery and three birth outcomes* were studied: delivery by C-section (ICD-10-CM: Z38.01), low birthweight (<2,500g), preterm birth (ICD-10-CM: O60, P073, P072), and admission to NICU. *HSU during pregnancy* included: all-cause hospitalizations, ED visits, and outpatient physician visits (excluding prenatal care visits). HSU were identified from any health encounters captured in health administrative databases (see Data sources above for details).

### Covariates

#### Pre-existing chronic diseases

We studied five different conditions. Asthma diagnosis in women was determined based on an administrative case definition of ≥1 hospitalization for asthma (International Classification of Diseases codes, ICD-9: 493 and ICD-10: J45, J46), or ≥2 outpatient visits for asthma in two consecutive years. This definition has been previously validated in Ontario with a sensitivity of 84% and a specificity of 77% [12]. Allergic rhinitis (ICD-9: 477 and ICD-10: J301-J304) and eczema (ICD-9: 691.8 and ICD-10: L20) were identified by any physician health services claim for these conditions [13]. Diagnosis of diabetes was identified from the Ontario Diabetes Database, a population-based disease registry using a validated algorithm to identify individuals with a new or pre-existing diagnoses of diabetes (ICD-9: 250 and ICD-10: E08-E13) [14]. Individuals were considered to have diabetes if they had at least one hospitalization or two physician service claims over a two-year period. Hypertension was identified from the Ontario Hypertension Database which used a validated case algorithm. Hypertension was said to be present if an individual had ≥1 hospital admission in two years or ≥2 OHIP claims with a hypertension diagnosis (ICD-9: 401x-405x; ICD-10: I10-I13, or I15) in three years [15].

In addition to women’s age at delivery, prenatal care visits, and existing chronic diseases outlined above, the following covariates were also included in the multivariable regression analyses. Socioeconomic status (SES) was measured by proxy, using the Ontario Marginalization Index with neighbourhood-level quintiles (ON-Marg, four domains: material deprivation, dependency, ethnic concentration, and residential instability) [16]. Based on each participant’s residence postal code, they were assigned a score from 1 (least marginalized) to 5 (most marginalized) for each domain. Residence was considered rural if the individual resided in a community with a population of ≤10,000 people, or urban if the opposite was true. Immigrant and refugee status were identified from the Refugees, and Citizenship Canada’s Permanent Resident Database. Comorbidities occurring during pregnancy such as pregnancy induced hypertension, preeclampsia, migraine, anxiety, mood disorders, and mental health conditions were identified from any health encounters for these conditions captured in CIHI-DAD, NACRS, OMHRS, or OHIP. Detailed ICD codes for these conditions were outlined in S1 Table. In-person and virtual prenatal care visits were identified and extracted from OHIP billings for outpatient physician visits in S2 Table.

### Statistical analysis

Baseline characteristics of the study populations were described with the frequency and proportion of categorical variables and the mean and standard deviation (SD) of numeric variables. Differences in baseline characteristics between pre-pandemic and pandemic periods were compared using standardized difference (SDiff) scores to provide a statistical measure of differences between groups that is not influenced by the large sample size [17,18]. A SDiff of >0.1 was considered a notable difference. We used multivariable logistic regression analyses with generalized estimating equations (GEE) to account for clustering and to estimate adjusted odds ratios (aOR) of mode of delivery and birth outcomes and negative binomial regression with GEE for adjusted rate ratios (aRR) of HSU during pregnancy. All aORs and aRRs were presented with 95% confidence intervals (CI). Additionally, we examined possible interactions between study periods and covariates and conducted sensitivity analyses by excluding those who were lab-tested positive for COVID (based on polymerase chain reaction testing) in the pandemic cohort. All regressions were adjusted for the covariates described above. All statistical analyses were conducted using SAS Enterprise Guide 7.1 (SAS Institute Inc., Cary, NC, USA). Ethics approval was waived from the Hospital for Sick Children Research Ethics Board (Toronto, Ontario, Canada) as this was a population-based study using only health administrative solely housed at ICES. The use of the data in this project is authorized under section 45 of Ontario’s Personal Health Information Protection Act (PHIPA) and does not require review by a Research Ethics Board.

## Results and discussion

### Population characteristics

This study included women who delivered a live birth before and/or during the COVID-19 pandemic (160,921 and 162,438, respectively). A total of 29,587 women had a live birth in both periods. Table 1 shows the percent distribution of characteristics of women who delivered in the two periods. In both periods, the average age of women was around 31 years, approximately a quarter of them were immigrants, and another 5% were refugees; there were no notable differences between periods based on ON-Marg, which were not statistically different between women who delivered in the pre-pandemic and pandemic periods. Albeit statistically non-significant, 9.3% and 7.3% of women who delivered in the pandemic and pre-pandemic periods, respectively, were without prenatal care visits.

**Table 1:** Percent distributions of characteristics of study populations.

		**Pre-pandemic**	**Pandemic**	**Standardized**
**Study population characteristics**		**(N = 160,921)**	**(N = 162,438)**	**difference^a^**
***Women’s demographic characteristics*:**				
Age at delivery (mean ± SD ^b^)		31.24 ± 5.10	31.55 ± 4.92	0.06
Without prenatal care visits		6.6%	8.7%	0.08
Rural residence		10.0%	10.0%	0.00
Immigrant status				
Immigrants		25.1%	24.3%	0.02
Refugees		5.1%	4.8%	0.02
Non-immigrants		69.8%	70.9%	0.02
***Women’s comorbidities*:**				
*Women’s pre-existing chronic diseases*:				
Asthma		16.7%	16.5%	0.01
Allergic rhinitis		36.3%	35.9%	0.01
Eczema		58.2%	58.0%	0.01
Diabetes		2.5%	4.1%	0.09
Hypertension		2.2%	2.1%	0.01
*Women’s pregnancy related comorbidities*:				
Pregnancy induced hypertension		4.4%	4.8%	0.02
Preeclampsia		4.2%	4.9%	0.03
*Women’s other comorbidities during pregnancy*:			
Migraine		3.3%	3.9%	0.03
Mood disorders		4.5%	4.6%	0.00
Anxiety		8.4%	10.2%	0.06
Any mental health conditions ^c^		12.1%	13.8%	0.05
***Women’s delivery characteristics*:**				
Days of stay at hospital (mean ± SD)		2.02 ± 2.00	1.90 ± 1.77	0.07
Women’s admission to ICU		0.3%	0.3%	0.00
		**Pre-pandemic**	**Pandemic**	**Standardized**
**Study population characteristics**		**(N = 160,921)**	**(N = 162,438)**	**difference** ^**a**^
***Birth characteristics*:**				
Newborn days of stay in hospital (mean ± SD)		2.36 ± 6.11	2.20 ± 5.51	0.03
NICU admission at birth		12.1%	11.9%	0.01
***Socioeconomic status based on residential neighbourhood characteristics*:**				
Neighbourhood income	Q1 (least)	21.5%	20.2%	0.03
	Q2	20.1%	20.0%	0.00
	Q3	21.3%	21.5%	0.01
	Q4	20.6%	20.9%	0.01
	Q5 (most)	16.3%	16.9%	0.02
	Missing	0.3%	0.4%	0.03
Material deprivation	Q1 (least)	21.5%	22.1%	0.01
	Q2	19.9%	20.2%	0.01
	Q3	18.4%	18.9%	0.01
	Q4	18.4%	18.2%	0.00
	Q5 (most)	20.6%	19.3%	0.03
	Missing	1.2%	1.3%	0.01
Dependency	Q1 (least)	33.1%	32.8%	0.01
	Q2	21.0%	21.0%	0.00
	Q3	16.6%	16.6%	0.00
	Q4	14.9%	15.0%	0.00
	Q5 (most)	13.2%	13.3%	0.00
	Missing	1.2%	1.3%	0.01
Ethnic concentration	Q1 (least)	13.1%	13.2%	0.00
	Q2	15.1%	15.4%	0.01
	Q3	17.0%	17.4%	0.01
	Q4	21.1%	21.5%	0.01
	Q5 (most)	32.6%	31.2%	0.03
	Missing	1.2%	1.3%	0.01
Residential instability	Q1 (least)	20.6%	20.8%	0.01
	Q2	18.1%	18.5%	0.01
	Q3	18.4%	18.7%	0.01
	Q4	18.4%	18.2%	0.01
	Q5 (most)	23.3%	22.4%	0.02
	Missing	1.2%	1.3%	0.01

^a^ Standardized difference of >0.1 is generally considered different.

^b^ Abbreviations: SD = standard deviations, ICU = intensive care unit, NICU = neonatal intensive care unit, Q1 = quintile 1, Q2 = quintile 2, Q3 = quintile3, Q4 = quintile 4, Q5 = quintile 5.

^c^ Includes: Substance-related and addictive disorders, schizophrenia spectrum and other psychotic disorders, mood disorders, anxiety, trauma/stressor-related or obsessive-compulsive disorder and related disorders and personality disorders.

The prevalence of pre-existing common chronic diseases such as asthma, allergic rhinitis, eczema, and hypertension among women in the two periods were similar (Table 1). The statistically non-significant prevalence of pre-existing diabetes was 4.1% and 2.5% in in women who delivered in the pandemic and pre-pandemic periods, respectively. There were also no statistical differences in pregnancy-related comorbidities (pregnancy induced hypertension, preeclampsia) and other comorbidities during pregnancy (migraine, mood disorders, anxiety, or other mental health conditions).

### Study outcomes

Table 2 shows the study outcomes by period. Women who delivered in the pandemic period had slightly higher and statistically different mean outpatient physician visits (6.63±5.84 versus 5.59±5.44), as well as lower but not statistically different mean ED visits (0.45±1.07 versus 0.56±1.19). About 1 in 5.5 women (18.3%) who delivered during the pandemic did not receive any COVID-19 vaccines, and only around 43% of them were vaccinated against COVID-19 before or during pregnancy. 8,521 women (5.2%) of the pandemic cohort lab-tested positive for COVID.

**Table 2:** Percent distribution of study outcomes by pre-pandemic and pandemic periods.

	Pre-pandemic	Pandemic	
Study outcomes	(N = 160,921)	(N = 162,438)	Sdiff ^a^^,^ ^b^
***Heath care utilization during pregnancy (mean ± SD)*:**			
All-cause hospitalizations	1.07 ± 0.33	1.07 ± 0.33	0.00
ED visits	0.56 ± 1.19	0.45 ± 1.07	0.10
Outpatient physician visits	5.59 ± 5.44	6.63 ± 5.84	0.19
***Mode of delivery*:**			
C-section delivery	29.8%	32.0%	0.05
***Birth outcomes*:**			
Birthweight (mean ± SD)	3,327.74 ± 558.65	3,330.32 ± 559.05	0.01
Low birthweight (<2,500 gm)	5.9%	5.9%	0.00
Preterm birth	7.0%	6.9%	0.00

^a^ Abbreviations: Sdiff = standardized difference, SD = standard deviations, ICU = intensive care unit, NICU = neonatal intensive care unit, ED = emergency department, C-section = Caesarean section.

^b^ Standardized difference of >0.1 is generally considered different.

None of the mode of delivery or birth outcomes were statistically different between the two periods. Mode of delivery and birth outcomes by women’s pre-existing chronic conditions and by pandemic periods are detailed in S3 Table. We also stratified outcomes by timing of COVID-19 vaccination and COVID-19 positivity in the pandemic cohort in S4 Table. Those who were vaccinated before/during pregnancy had lower all-cause ED visits (0.41±1.00 versus 0.55±1.26, Sdiff = 0.13). Those who lab-tested positive for COVID-19 had significantly more ED visits (0.67±1.18 versus 0.44±1.07, Sdiff = 0.21) and all-cause hospitalization (0.12±0.44 versus 0.07±0.32, Sdiff = 0.14), but significantly less physician visits (6.55±5.78 versus 8.22±6.69, Sdiff = 0.27).

#### C-section delivery

**Fig 2:**
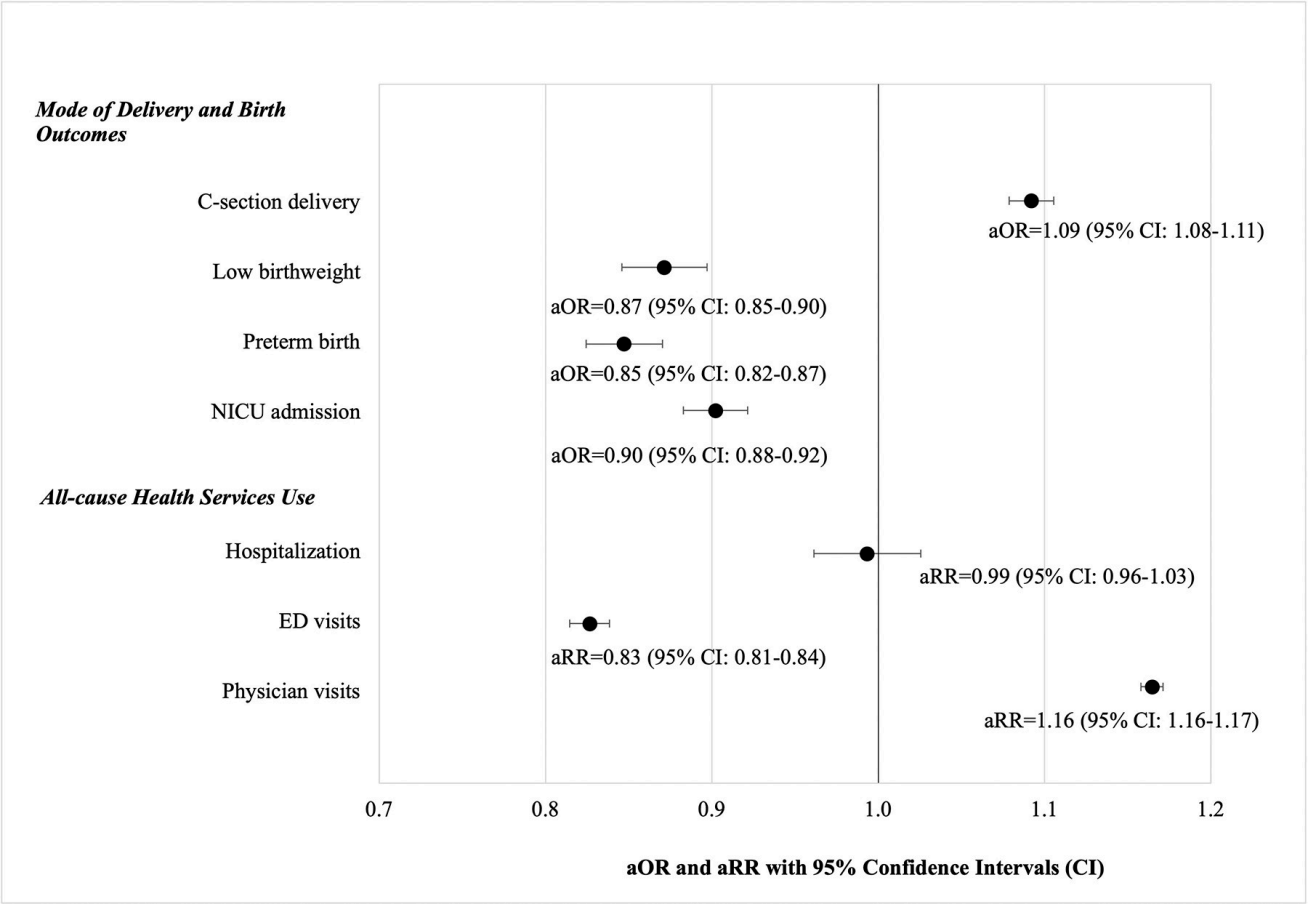
Adjusted odds ratios (aOR) of mode of delivery and birth outcomes and adjusted rate ratios (aRR) of health services use.

In the multivariable logistic regression analysis, the adjusted odds of C-section delivery in the pandemic period was 9% higher than the pre-pandemic period (aOR = 1.09, 95%CI: 1.08–1.11) after adjusting for covariates (Table 3, Fig 2). Table 3 also shows that immigrants tended to have significantly higher odds of C-section delivery. Women who experienced comorbidities during pregnancy such as migraine, mental health conditions, pregnancy induced hypertension, and preeclampsia had statistically significant increased odds of C-section delivery. Those with pre-existing chronic conditions such as diabetes and hypertension had over 50% increased odds of C-section delivery (aOR = 1.55, 95%CI: 1.44–1.66 and aOR = 1.52, 95%CI: 1.37–1.70, respectively).

**Table 3:** Adjusted odds ratios of mode of delivery and birth outcomes from multivariable logistic regressions.

		C-section Delivery ^a^				Low Birthweight				Preterm Birth				NICU Admission			
Covariates		aOR	95% CI			aOR	95% CI			aOR	95% CI			aOR	95% CI		
***Primary exposure*:**																	
Pandemic		1.09	1.08	-	1.11	0.87	0.85	-	0.90	0.85	0.82	-	0.87	0.90	0.88	-	0.92
Pre-pandemic (reference)																	
***Demographics*:**																	
Women’s age at delivery																	
36–50		1.93	1.88	-	1.97	1.02	0.98	-	1.07	1.20	1.15	-	1.25	1.04	1.00	-	1.07
29–35		1.32	1.29	-	1.34	0.86	0.83	-	0.89	0.95	0.91	-	0.98	0.91	0.89	-	0.94
13–28 (reference)																	
Immigrant status																	
Immigrants		1.06	1.03	-	1.08	1.29	1.24	-	1.34	1.18	1.13	-	1.22	1.01	0.98	-	1.04
Refugees		1.10	1.06	-	1.14	0.97	0.90	-	1.04	1.07	1.00	-	1.14	0.93	0.89	-	0.99
Non-immigrants (reference)																
Prenatal care visits (continuous)		1.02	1.02	-	1.02	0.92	0.92	-	0.92	0.90	0.90	-	0.91	0.96	0.96	-	0.96
Rural residence		1.03	1.00	-	1.06	0.91	0.86	-	0.97	0.92	0.87	-	0.97	0.78	0.75	-	0.82
Marginalization index (Q1 as reference)																	
Deprivation	Q2	1.00	0.98	-	1.03	1.10	1.04	-	1.15	1.03	0.99	-	1.08	1.08	1.05	-	1.12
	Q3	1.02	1.00	-	1.05	1.17	1.12	-	1.23	1.14	1.09	-	1.20	1.16	1.12	-	1.21
	Q4	1.05	1.02	-	1.07	1.16	1.10	-	1.22	1.13	1.07	-	1.18	1.21	1.16	-	1.25
	Q5	1.08	1.05	-	1.11	1.30	1.23	-	1.37	1.28	1.22	-	1.35	1.33	1.28	-	1.38
		**C-section Delivery** ^**a**^				**Low Birthweight**				**Preterm Birth**				**NICU Admission**			
**Covariates**	** **			**aOR**	**95% CI**			**aOR**	**95% CI**			**aOR**	**95% CI**			**aOR**	**95% CI**		
Dependency	Q2	0.96	0.94	-	0.98	0.98	0.94	-	1.03	0.99	0.96	-	1.04	0.92	0.89	-	0.95
	Q3	0.96	0.93	-	0.98	1.03	0.98	-	1.08	1.02	0.97	-	1.06	0.95	0.92	-	0.99
	Q4	0.97	0.94	-	0.99	1.05	0.99	-	1.10	1.05	1.00	-	1.10	0.94	0.90	-	0.97
	Q5	0.97	0.95	-	1.00	1.08	1.01	-	1.14	1.04	0.99	-	1.10	0.98	0.94	-	1.02
Ethnic concentration	Q2	0.99	0.96	-	1.02	0.95	0.89	-	1.01	1.01	0.95	-	1.06	0.99	0.95	-	1.03
	Q3	0.95	0.92	-	0.98	1.04	0.98	-	1.11	1.03	0.97	-	1.09	0.97	0.92	-	1.01
	Q4	0.95	0.92	-	0.98	1.19	1.11	-	1.27	1.02	0.96	-	1.08	0.96	0.92	-	1.00
	Q5	0.96	0.93	-	0.99	1.45	1.36	-	1.55	1.14	1.08	-	1.21	0.99	0.95	-	1.04
Instability	Q2	1.03	1.00	-	1.06	0.95	0.90	-	1.00	0.98	0.93	-	1.03	1.00	0.96	-	1.03
	Q3	1.03	1.01	-	1.06	1.00	0.95	-	1.05	1.00	0.95	-	1.04	1.00	0.96	-	1.04
	Q4	1.04	1.02	-	1.07	1.01	0.96	-	1.07	1.02	0.97	-	1.07	1.02	0.98	-	1.06
	Q5	1.07	1.04	-	1.10	1.07	1.02	-	1.13	1.03	0.98	-	1.08	0.98	0.95	-	1.02
***Pre-existing chronic conditions*:**																	
Asthma only		1.04	0.98	-	1.11	1.16	1.03	-	1.31	1.34	1.21	-	1.49	1.17	1.08	-	1.28
Allergic rhinitis only		0.95	0.92	-	0.99	0.97	0.91	-	1.04	1.03	0.97	-	1.10	1.00	0.95	-	1.05
Eczema only		0.93	0.91	-	0.95	0.99	0.95	-	1.03	1.05	1.01	-	1.09	0.99	0.96	-	1.02
Diabetes only		1.55	1.44	-	1.66	1.24	1.08	-	1.44	1.95	1.74	-	2.20	2.14	1.95	-	2.34
Hypertension only		1.52	1.37	-	1.70	1.91	1.62	-	2.26	1.86	1.58	-	2.18	1.49	1.29	-	1.71
>1 of the above conditions		1.05	1.03	-	1.08	1.11	1.06	-	1.15	1.28	1.23	-	1.33	1.16	1.13	-	1.19
None of these conditions (reference)																
***Comorbidities during pregnancy*:**																	
Migraine		1.10	1.06	-	1.14	1.00	0.92	-	1.08	1.03	0.96	-	1.11	1.05	0.99	-	1.11
Mental health conditions		1.13	1.11	-	1.15	1.35	1.29	-	1.40	1.32	1.27	-	1.37	1.51	1.46	-	1.55
Pregnancy induced hypertension		1.18	1.14	-	1.22	1.23	1.15	-	1.32	1.05	0.98	-	1.11	1.32	1.26	-	1.39
Preeclampsia		1.62	1.57	-	1.67	4.13	3.92	-	4.35	4.07	3.88	-	4.27	2.65	2.54	-	2.77

^a^ Abbreviations: C-section = Caesarean section, aOR = adjusted odds ratio, CI = confidence intervals, Q1 = quintile 1, Q2 = quintile 2, Q3 = quintile3, Q4 = quintile 4, Q5 = quintile 5.

All aORs and aRRs were adjusted for other covariates: Mother’s age at delivery, immigrant status, rural residence, prenatal care visits, marginalization index, pre-existing chronic conditions, and comorbidities during pregnancy.

#### Birth outcomes

The adjusted odds of low birthweight and preterm birth were 13% and 15% lower in the pandemic period, respectively (aOR = 0.87, 95%CI: 0.85–0.90 and aOR = 0.85, 95%CI: 0.82–0.87, respectively). Similarly, the adjusted odds of NICU admission were 10% lower in the pandemic period (aOR = 0.90, 95%CI: 0.88–0.92).

There were a few significant predictors for the above outcomes. Compared to non-immigrants, immigrants had higher odds of preterm births (aOR = 1.18, 95%CI: 1.13–1.22) and their babies had 29% higher odds of having low birthweight (aOR = 1.29, 95%CI: 1.24–1.34). Women with higher number of prenatal care visits were associated with lower odds of having low birthweight babies and having their babies admitted to NICU. Babies born to women who experienced preeclampsia during pregnancy had 4-fold increased odds of having low birthweight or preterm birth (aOR = 4.13, 95%CI: 3.92–4.35 and aOR = 4.07, 95%CI: 3.88–4.27, respectively), and increased odds of NICU admissions (aOR = 2.65, 95%CI: 2.54–2.77). Similarly, babies born to women with prevalent asthma, diabetes, and hypertension had significantly higher odds of having low birthweight, preterm birth, and NICU admissions.

#### Health services use

Table 4 and Fig 2 show the adjusted rate ratios (aRR) of all-cause hospitalization, ED visits, and physician visits comparing the pandemic period to the pre-pandemic period. While there was no statistically significant difference in the aRR of all-cause hospitalization, there was a statistically significant 17% reduction in ED visits but a 16% increase in physician visits in the pandemic period (aRR = 0.83, 95%CI: 0.81–0.84 and aRR = 1.16, 95%CI: 1.16–1.17, respectively).

**Table 4:** Adjusted rate ratios of all-cause health services use from multivariable negative binomial regressions.

		**Hospitalization**				**ED Visits**				**Physician Visits**			
**Covariates**	** **	**aRR** ^**a**^	**95% CI**			**aRR**	**95% CI**			**aRR**	**95% CI**		
***Primary exposure*:**													
Pandemic		0.99	0.96	-	1.03	0.83	0.81	-	0.84	1.16	1.16	-	1.17
Pre-pandemic (reference)															
***Demographics*:**													
Women’s age at delivery													
36–50		0.95	0.90	-	0.99	0.58	0.57	-	0.60	1.03	1.03	-	1.04
29–35		0.80	0.77	-	0.83	0.62	0.61	-	0.63	1.09	1.08	-	1.10
13–28 (reference)													
Immigrant status													
Immigrants		0.94	0.90	-	0.99	0.97	0.95	-	0.99	1.27	1.26	-	1.28
Refugees		1.07	0.99	-	1.15	1.13	1.10	-	1.17	1.19	1.17	-	1.20
Non-immigrants (reference)													
Prenatal care visits (continuous)		1.02	1.02	-	1.02	1.02	1.02	-	1.02	1.00	1.00	-	1.00
Rural residence		1.01	0.95	-	1.08	1.56	1.52	-	1.60	0.73	0.72	-	0.74
Marginalization index (Q1 as reference)													
Deprivation	Q2	1.04	0.99	-	1.09	1.09	1.06	-	1.11	1.04	1.03	-	1.05
	Q3	1.02	0.97	-	1.08	1.15	1.12	-	1.18	1.06	1.05	-	1.07
	Q4	1.04	0.98	-	1.10	1.27	1.24	-	1.30	1.02	1.01	-	1.03
	Q5	1.16	1.10	-	1.23	1.53	1.49	-	1.57	0.95	0.94	-	0.96
Dependency	Q2	0.96	0.91	-	1.01	0.92	0.90	-	0.94	0.99	0.98	-	1.00
	Q3	0.96	0.91	-	1.01	0.91	0.89	-	0.93	0.97	0.96	-	0.98
	Q4	1.00	0.95	-	1.06	0.94	0.92	-	0.97	0.97	0.96	-	0.98
	Q5	0.99	0.93	-	1.05	0.98	0.95	-	1.01	0.98	0.97	-	1.00
Ethnic concentration	Q2	0.99	0.93	-	1.05	0.90	0.87	-	0.92	1.10	1.09	-	1.12
	Q3	1.07	1.00	-	1.15	0.82	0.80	-	0.84	1.25	1.24	-	1.27
	Q4	1.15	1.08	-	1.23	0.77	0.74	-	0.79	1.43	1.41	-	1.45
	Q5	1.18	1.10	-	1.26	0.76	0.74	-	0.78	1.78	1.76	-	1.81
Instability	Q2	1.06	1.00	-	1.12	1.00	0.98	-	1.03	0.95	0.94	-	0.96
	Q3	1.07	1.01	-	1.14	1.01	0.99	-	1.04	0.98	0.97	-	0.99
	Q4	1.19	1.12	-	1.26	1.03	1.00	-	1.06	0.97	0.96	-	0.98
	Q5	1.31	1.24	-	1.38	1.05	1.02	-	1.07	1.00	0.99	-	1.01
		**Hospitalization**				**ED Visits**				**Physician Visits**			
**Covariates**	** **	**aRR** ^**a**^	**95% CI**			**aRR**	**95% CI**			**aRR**	**95% CI**		
***Pre-existing chronic conditions*:**													
Asthma only		1.17	1.04	-	1.32	1.33	1.26	-	1.41	1.02	1.00	-	1.05
Allergic rhinitis only		1.03	0.96	-	1.11	1.09	1.05	-	1.12	1.05	1.03	-	1.06
Eczema only		1.07	1.02	-	1.12	1.08	1.06	-	1.10	1.02	1.01	-	1.03
Diabetes only		1.57	1.34	-	1.85	1.27	1.17	-	1.38	1.73	1.69	-	1.78
Hypertension only		1.54	1.22	-	1.94	1.28	1.15	-	1.43	1.38	1.32	-	1.43
>1 of the above conditions		1.32	1.26	-	1.37	1.30	1.28	-	1.33	1.20	1.19	-	1.21
None of these conditions (reference)													
***Comorbidities during pregnancy*:**													
Migraine		1.49	1.39	-	1.61	1.59	1.54	-	1.65	1.53	1.51	-	1.55
Mental health conditions		1.95	1.87	-	2.03	1.80	1.76	-	1.84	1.71	1.70	-	1.73
Pregnancy induced hypertension		1.78	1.68	-	1.89	1.12	1.08	-	1.16	1.12	1.10	-	1.13
Preeclampsia		1.91	1.80	-	2.02	1.25	1.21	-	1.29	1.27	1.25	-	1.29

^a^ Abbreviations: aRR = adjusted rate ratio; CI = confidence intervals; Q1 = quintile 1, Q2 = quintile 2, Q3 = quintile3, Q4 = quintile 4, Q5 = quintile 5.

Table 4 also showed that refugees, compared to non-immigrants, tended to have higher RR of ED and physician visits (aRR = 1.13, 95%CI: 1.10–1.17 and aRR = 1.19, 95%CI:1.17–1.20, respectively). Women with pre-existing chronic conditions or who experienced comorbidities during pregnancy were all associated with significantly increased aRR in all HSU.

Possible interactions between study periods and covariates were also examined. None were found to be statistically significant nor clinically relevant. Thus, these findings were not presented here.

## Discussion

This current study used large population-based administrative data to compare delivery and birth outcomes of 160,921 births that occurred in the 21 months prior to the COVID-19 pandemic to 162,438 births that occurred in the 21 months during the pandemic in Ontario, Canada. To our knowledge, this study is the first that quantified the impact of the pandemic in this population in Canada, and with the longest and most recent follow-up data. The results of our adjusted multivariable regression analysis suggested a 9% (OR = 1.09) significantly higher odds of C-section delivery during the pandemic compared to pre-pandemic. Our data also showed that during the pandemic, healthcare utilization in pregnant women was lower compared to the pre-pandemic period. Several predictors, described in the following paragraphs, may have contributed to these outcomes.

We found that immigrant women had significantly higher odds of C-section, compared to non-immigrant women. Similarly, refugees tended to have higher rate ratios of ED and physician visits during their pregnancy. Pregnant women with prevalent diabetes or hypertension, and those who experienced morbidities during pregnancy, had higher odds of C-section and higher all-cause healthcare utilization compared to those without these conditions. Others have found that the increase in C-section rates may reflect the efforts of obstetricians to provide the “best services” to their patients in the face of constantly changing guidelines during the pandemic regarding the safest delivery method for the mother, baby, and provider [19–21]. These “best services” during the pandemic may have also contributed to the lower rates of low birthweight and preterm birth.

Despite international recommendations [22], COVID-19 vaccine acceptance among pregnant women was much lower than that in the general population [23,24]. One reason for the reduced rate of COVID-19 vaccine uptake may be pregnant women’s lower confidence in the safety and efficacy of COVID-19 vaccines in both mothers and babies, likely due to minimal limited evidence on these populations at the time [25–27]. Similarly, in our pandemic cohort of pregnant women, the majority (58%) were not vaccinated before or during pregnancy. Fortunately, the lab-tested COVID positivity rate was relatively low (5%). We performed sensitivity analyses stratifying the mode of delivery and birth outcomes in the pandemic cohort by vaccinated versus not, and COVID-19 positive versus negative. Similar to findings of a systematic review and meta-analysis that included 11 studies and 756,098 pregnant women [28], while there was a slightly lower number of ED visits in pregnant women who were vaccinated before/during pregnancy, we did not find other statistically significant and clinically relevant differences in delivery and birth outcomes in the vaccinated versus non-vaccinated pregnant women (see S4 Table).

Results from an online international cross-sectional survey with participants from developed countries (USA, Ireland, and the UK) [9] suggested the lack of access to antenatal care coupled with reduced family/social support potentially intensified pregnancy specific stress. Using population-based health administrative data that captured health care encounters associated with migraine, anxiety, and any mental health conditions—possible outcomes of pregnancy specific stress—our study showed a slightly higher level of these conditions among pregnant women during the pandemic, however, they were not statistically different compared to the pre-pandemic cohort.

While our study included most recent deliveries that occurred during the COVID-19 pandemic from July 2020 to March 2022, there are several limitations to this study. First, we may have two instances of statistical analysis bias, namely when we excluded 1) 99 women >50 years which constituted <0.06% of the study population and 2) those with incomplete observed data due to loss of OHIP coverage, attributable to migration to other provinces or countries, comprising of <3% of the population. However, these excluded numbers were small, thus we do not expect significant statistical bias by excluding these groups. Next, we were unable to include gestational diabetes in our analysis as they were not included in the Ontario Diabetes Database. Using a published health administrative data algorithm, we identified only about 4% of our pandemic cohort who had gestational diabetes, which was less than 50% of that found in the pre-pandemic cohort. Hence, we were unable to use health administrative data to capture gestational diabetes as a risk factor. On the other hand, the strength of population-based data from a single payer organized health system with linked mother-baby records and healthcare utilization allowed for complete capture with a large sample size, and high power, which was necessary to compare outcomes in the pre-pandemic and pandemic periods.

## Conclusion

In Ontario, Canada, there were approximately 160,000 births before and during the pandemic. During the COVID-19 pandemic, there was a decline in ED visits as well as an increase in physician office visits amongst pregnant women. Identifying those who are at risk for morbidities (e.g., anxiety, mental health conditions) and pregnancy related complications (e.g., pregnancy-induced hypertension, preeclampsia), those with a pre-existing chronic disease (e.g., diabetes, hypertension, asthma, and other allergic conditions), or those who are an immigrant/refugee, and then ensuring that these individuals have access to overall health care and prenatal care may prevent adverse pregnancy and birth outcomes.

## Supporting information

S1 Table International Classification of Diseases (ICD) codes for comorbidities during pregnancy. (DOCX)

S2 Table Ontario Health Insurance Plan (OHIP) fee codes associated with prenatal visits. (DOCX)

S3 Table Mode of delivery and birth outcomes by pregnant women’s pre-existing chronic conditions and by pre-pandemic and pandemic periods. (DOCX)

S4 Table Mode of delivery and birth outcomes by pregnant women’s vaccination status and COVID-19 infection during the pandemic period. (DOCX)
