# Supplementary material for: Mode of delivery and birth outcomes before and during COVID-19 –A population-based study in Ontario, Canada
Source: PLoS One. 2024 May 10;19(5):e0303175. doi: 10.1371/journal.pone.0303175 (PMC11086824; doi:10.1371/journal.pone.0303175)
Supplement: S1 Table — (DOCX) [file pone.0303175.s001.docx]

**S1 Table.** International Classification of Diseases (ICD) codes for comorbidities during pregnancy.

| **Comorbidity** | **ICD-10-CA (CIHI-DAD, NACRS)** | **DXCODE (OHIP)** | **ICD-10-CM (OMHRS)** | **ICD-9-CM (OMHRS DSM5)** |
| --- | --- | --- | --- | --- |
| Gestational diabetes | O244 | N/A | N/A | N/A |
| Pre-eclampsia | O14 | 642 | N/A | N/A |
| Pregnancy induced hypertension | O13 | N/A | N/A | N/A |
| Migraine | G43909 | 346 | N/A | N/A |
| *Mental health conditions* |  |  |  |  |
| Substance-related and addictive disorders | F10-19, F55 | 291, 292, 303, 304 | F10.x-F19.x, Z72.0. | 291.x, 292.x, 303.x, 304.x, 305.x. |
| Schizophrenia spectrum and other psychotic disorders | F06.0-2, F20, F22-F29, F53.1 | 295, 297, 298 | F20.81, F20.9, F22, F23, F25, F06.0/1/2, F28, F29. | 293.81, 293.82, 295.x, 297.x, 298.x |
| Mood disorders | F06.3, F30.x-F34.x, F38.x, F39.x, F53.0 | 296, 311, 309 | F06.3, F31, F32, F33, F34. ​ | 293.83, 296.x, 300.4x, 301.13, 311.x, 625.4 |
| Anxiety | F06.4, F40, F41, F93.0-2, F94.0 | 300 | F06.4, F40.0x, F40.1x. F40.2x, F41.0x/1x, F41.8x/9x, F93.0, F94.0. | 293.84, 300.0x, 300.2x, 309.21, 313.23 |
| Trauma/stressor-related disorders | F43.x, F94.1, F94.2 | N/A | F43.0, F43.1. F43.2, F43.8/9, F94.1/2. | 308.3x, 309, 313.89 |
| Obsessive-compulsive disorder and related disorders | F42.x, F45.2, F63.3 | N/A | F06.8, F42.2x, F42.3, F42.4, F42.8, F42.9, F45.2, F63.3 | 300.3x, 300.7x, 312.39, 698.4x |
| Personality disorders | F07, F21, F60, F61, F62, F68, F69 | 301, 306, 307 | F07.x, F21, F60. | 301, 310.1 |

Abbreviations: ICD=International Classification of Diseases; CIHI-DAD= Canadian Institute for Health Information Discharge Abstract Database; NACRS= National Ambulatory Care Reporting System; DX=diagnosis code; OHIP=Ontario Health Insurance Plan; OMHRS= Ontario Mental Health Reporting System; DSM= Diagnostic and Statistical Manual of Mental Disorders.
