# Supplementary material for: Mode of delivery and birth outcomes before and during COVID-19 –A population-based study in Ontario, Canada
Source: PLoS One. 2024 May 10;19(5):e0303175. doi: 10.1371/journal.pone.0303175 (PMC11086824; doi:10.1371/journal.pone.0303175)
Supplement: S2 Table — (DOCX) [file pone.0303175.s002.docx]

**S2 Table.** Ontario Health Insurance Plan (OHIP) fee codes associated with prenatal visits.

| **OHIP Fee Code** | **Description** |
| --- | --- |
| A005 | Consultation |
| A006 | Repeat consultation |
| A204 | Partial assessment |
| A205 | Consultation |
| A206 | Repeat consultation |
| A665 | Prenatal consultation |
| A920 | Medical management of early pregnancy, initial service |
| A921 | Medical management of early or ectopic pregnancy, follow-up visit |
| P002 | High risk prenatal assessment |
| P003 | General assessment (major prenatal visit) |
| P004 | Minor prenatal assessment |
| P005 | Antenatal preventative health assessment |
| Q606 | General assessment (major prenatal visit) |
| Q607 | Minor assessment (follow-up prenatal visit) |

Note: Prenatal care visits were defined with the fee codes above and with physician speciality in Family Practice/General Practitioner (FP/GP) or Obstetrics and Gynecology; the visits were limited to one visit per person per doctor specialty per day.
