# Supplementary material for: Mode of delivery and birth outcomes before and during COVID-19 –A population-based study in Ontario, Canada
Source: PLoS One. 2024 May 10;19(5):e0303175. doi: 10.1371/journal.pone.0303175 (PMC11086824; doi:10.1371/journal.pone.0303175)
Supplement: S3 Table — (DOCX) [file pone.0303175.s003.docx]

**S3 Table.** Mode of delivery and birth outcomes by pregnant women’s pre-existing chronic conditions and by pre-pandemic and pandemic periods.

|  | **Asthma** | | **Allergic Rhinitis** | | **Eczema** | | **Diabetes** | | **Hypertension** | | **Overall N (%)** | |
| --- | --- | --- | --- | --- | --- | --- | --- | --- | --- | --- | --- | --- |
|  | **Before** | **During** | **Before** | **During** | **Before** | **During** | **Before** | **During** | **Before** | **During** | **Before** | **During** |
| ***Mode of Delivery, Mother Outcomes:*** |  |  |  |  |  |  |  |  |  |  |  |  |
| C-section | 30.6% | 32.7% | 30.3% | 32.4% | 29.4% | 31.5% | 48.1% | 45.8% | 47.0% | 49.2% | 47,954 (29.8%) | 51,495 (32.0%) |
| PIH | 5.4% | 5.7% | 4.7% | 5.2% | 4.7% | 5.1% | 9.5% | 8.6% | 20.7% | 23.3% | 7,081 (4.4%) | 7,724 (4.8%) |
| Pre-eclampsia | 4.9% | 5.8% | 4.5% | 5.3% | 4.4% | 5.1% | 9.8% | 8.8% | 20.0% | 23.2% | 6,759 (4.2%) | 7,885 (4.9%) |
| Migraine | 3.8% | 4.5% | 3.8% | 4.5% | 3.5% | 4.0% | 4.5% | 4.6% | 4.6% | 5.0% | 5,310 (3.3%) | 6, 275 (3.9%) |
| Mood disorders | 6.5% | 7.0% | 5.2% | 5.3% | 5.2% | 5.3% | 6.8% | 6.0% | 5.7% | 6.2% | 7,241 (4.5%) | 7,402 (4.6%) |
| Anxiety | 11.2% | 13.7% | 10.2% | 12.6% | 9.7% | 11.9% | 10.6% | 11.6% | 11.0% | 14.4% | 13,517 (8.4%) | 16,414 (10.2%) |
| Any mental health conditions ^a^ | 16.0% | 18.7% | 14.1% | 16.4% | 13.6% | 15.8% | 15.8% | 16.3% | 15.6% | 19.6% | 19,471 (12.1%) | 22,207 (13.8%) |
| At least one COVID-19 vaccine dose | N/A | 80.1% | N/A | 82.2% | N/A | 80.9% | N/A | 84.6% | N/A | 84.1% | N/A | 131,473 (81.7%) |
| ***Baby Outcomes:*** |  |  |  |  |  |  |  |  |  |  |  |  |
| % Low birthweight | 6.1% | 5.8% | 5.8% | 5.6% | 5.7% | 5.6% | 8.5% | 8.3% | 12.0% | 12.3% | 9,333 (5.8%) | 9,421 (5.8%) |
| % Preterm birth ^b^ | 7.8% | 7.6% | 7.2% | 7.1% | 7.1% | 7.1% | 16.1% | 13.9% | 14.2% | 15.5% | 11,264 (7.0%) | 11,208 (6.9%) |
| NICU admission | 13.5% | 13.2% | 12.4% | 12.1% | 12.2% | 12.1% | 27.9% | 21.8% | 20.7% | 21.4% | 19,471 (12.1%) | 19,330 (11.9%) |
| % with congenital anomalies | 6.2% | 6.0% | 5.9% | 5.7% | 5.8% | 5.8% | 7.9% | 6.8% | 6.1% | 6.9% | 9172 (5.7%) | 9,097 (5.6%) |

^a^ Includes: Substance-related and addictive disorders, schizophrenia spectrum and other psychotic disorders, mood disorders, anxiety, trauma/stressor-related disorders, or obsessive-compulsive disorders and related disorders, and personality disorders.

^b^ Defined using diagnosis codes O60, P073, and P072 in the newborn records.
