# Supplementary material for: Mode of delivery and birth outcomes before and during COVID-19 –A population-based study in Ontario, Canada
Source: PLoS One. 2024 May 10;19(5):e0303175. doi: 10.1371/journal.pone.0303175 (PMC11086824; doi:10.1371/journal.pone.0303175)
Supplement: S4 Table — (DOCX) [file pone.0303175.s004.docx]

**S4 Table.** Mode of delivery and birth outcomes by pregnant women’s vaccination status and COVID-19 infection during the pandemic period.

|  | **COVID-19 Vaccination** | | | |  | **COVID-19 Positivity** | | |
| --- | --- | --- | --- | --- | --- | --- | --- | --- |
|  | **Not vaccinated** | **Before/during pregnancy** |  | **Post pregnancy** |  | **Positive** | **Negative** |  |
|  | **(N=29,700)** | **(N=69,025)** |  | **(N=63,713)** |  | **(N=8,521)** | **(N=153,917)** |  |
| **Study population characteristics** | **18.30%** | **42.50%** | **Sdiff ^a, b^** | **39.20%** | **Sdiff** | **5.0%** | **95.0%** | **Sdiff** |
| ***Delivery characteristics:*** | | | | |  |  |  |  |
| Days of stay at hospital (mean ± SD): | 1.81 ± 1.61 | 1.91 ± 1.74 | 0.059 | 1.93 ± 1.86 | 0.059 | 1.98 ± 2.17 | 1.90 ± 1.74 | 0.041 |
| Women’s admission to ICU | 0.4% | 0.3% | 0.028 | 0.3% | 0.015 | 0.9% | 0.3% | 0.074 |
| ***Birth characteristics:*** |  |  |  |  |  |  |  |  |
| Newborn days of stay in hospital | 2.28 ± 6.38 | 2.04 ± 4.41 | 0.044 | 2.32 ± 6.12 | 0.044 | 2.28 ± 5.61 | 2.19 ± 5.51 | 0.016 |
| NICU admission at birth | 12.3% | 11.2% | 0.035 | 12.5% | 0.005 | 12.8% | 11.8% | 0.030 |
| ***Heath care utilization during pregnancy (mean ± SD):*** | | | | |  |  |  |  |
| All-cause hospitalizations | 0.08 ± 0.35 | 0.07 ± 0.32 | 0.029 | 0.07 ± 0.31 | 0.027 | 0.12 ± 0.44 | 0.07 ± 0.32 | 0.138 |
| ED visits | 0.55 ± 1.26 | 0.41 ± 1.00 | 0.130 | 0.45 ± 1.06 | 0.089 | 0.67 ± 1.18 | 0.44 ± 1.07 | 0.207 |
| Outpatient physician visits | 5.81 ± 5.72 | 6.90 ± 5.81 | 0.189 | 6.74 ± 5.90 | 0.160 | 6.55 ± 5.78 | 8.22 ± 6.69 | 0.267 |
| ***Mode of delivery:*** |  |  |  |  |  |  |  |  |
| C-section delivery | 29.0% | 32.8% | 0.081 | 32.5% | 0.075 | 33.2% | 31.9% | 0.029 |
| ***Birth outcomes:*** |  |  |  |  |  |  |  |  |
| Birthweight (mean ± SD) | 3,344.43 ± 569.51 | 3,330.41 ± 544.03 | 0.025 | 3,323.65 ± 569.96 | 0.025 | 3,300.16 ± 549.86 | 3,331.99 ± 559.51 | 0.057 |
| Low birthweight (<2,500 gm) | 6.0% | 5.3% | 0.029 | 6.1% | 0.005 | 6.0% | 5.8% | 0.010 |
| Preterm birth | 7.0% | 6.5% | 0.022 | 7.4% | 0.015 | 7.6% | 6.9% | 0.027 |

^a^ Abbreviations: Sdiff=standardized difference, SD=standard deviations, ICU=intensive care unit, NICU=neonatal intensive care unit, ED=emergency department, C-section=Caesarean section.

^b^ Standardized difference of >0.1 is generally considered different.
